# Nevus Unius Lateris With Epidermolytic Hyperkeratosis Managed With Radiofrequency Ablation: A Case Report

**DOI:** 10.7759/cureus.113773

**Published:** 2026-08-01

**Authors:** Joel Jack, Divya Raviprakash, Leena Joseph, Murugan Sundaram

**Affiliations:** 1 Dermatology, Sri Ramachandra Institute of Higher Education and Research, Chennai, IND; 2 Pathology, Sri Ramachandra Institute of Higher Education and Research, Chennai, IND

**Keywords:** cutaneous mosaicism, epidermal nevus syndrome, epidermolytic hyperkeratosis, genetic counseling, hamartoma, nevus unius lateris, radiofrequency ablation, verrucous epidermal nevus

## Abstract

Nevus unius lateris is a rare, systematized clinical variant of verrucous epidermal nevus, characterized by extensive unilateral verrucous lesions following the lines of Blaschko. While most epidermal nevi are localized, the systematized form often raises concerns for epidermal nevus syndrome, which involves associated multisystem abnormalities in the skeletal, ocular, and neurological systems.

We present the case of an 18-year-old male with extensive, hyperpigmented, verrucous plaques involving the left side of his trunk, upper limb, and lower limb without associated extracutaneous abnormalities. The lesions had been present since early childhood and showed significant progression following puberty. A comprehensive systemic evaluation, including orthopedic and ophthalmological consultations, revealed no associated internal anomalies. Histopathological examination of a biopsy specimen confirmed the diagnosis of a verrucous epidermal nevus with prominent epidermolytic hyperkeratosis. The patient was managed with multiple sessions of radiofrequency ablation for selected lesions, with satisfactory cosmetic and symptomatic improvement.

This case underscores the importance of a thorough systemic workup in patients with extensive epidermal nevi and highlights the role of ablative therapies in managing the cutaneous manifestations of this rare condition.

## Introduction

Verrucous epidermal nevi (VEN) are cutaneous hamartomas originating from the embryonic ectoderm that are typically present at birth or develop during early childhood. These lesions are classified based on their clinical appearance and extent. Nevus unius lateris represents a specific and rare clinical subtype where the lesions are particularly extensive and strictly unilateral [1]. These nevi follow the lines of Blaschko, which represent the pathways of epidermal cell migration during embryogenesis. Lesions usually follow a benign course and may enlarge, thicken, and stabilize by puberty [2]. However, only a limited number of cases have been reported in the literature [3]. Verrucous epidermal nevus may occur as part of epidermal nevus syndrome with central nervous system, ocular, or skeletal abnormalities; however, the epidermolytic variant is typically not associated with extracutaneous manifestations [4]. Rare cases of malignant transformation of epidermal nevi into basal cell carcinoma and squamous cell carcinoma have been reported [5]. We report an 18-year-old male with an extensive isolated presentation of nevus unius lateris with epidermolytic hyperkeratosis, highlighting the importance of excluding epidermal nevus syndrome and the role of radiofrequency ablation in management.

## Case presentation

An 18-year-old male presented to the dermatology outpatient department with a history of raised, dark-colored skin lesions over the left side of his body since the age of five. The lesions initially appeared on the left thigh and gradually progressed over 13 years to involve the left upper limb, chest, abdomen, back, and genital region. The patient reported that the lesions became more prominent and thicker following the onset of puberty. There was no history of itching, discharge, or pain associated with the plaques. Furthermore, the patient denied any history of seizures, developmental delays, or visual disturbances.

**Figure 1:**
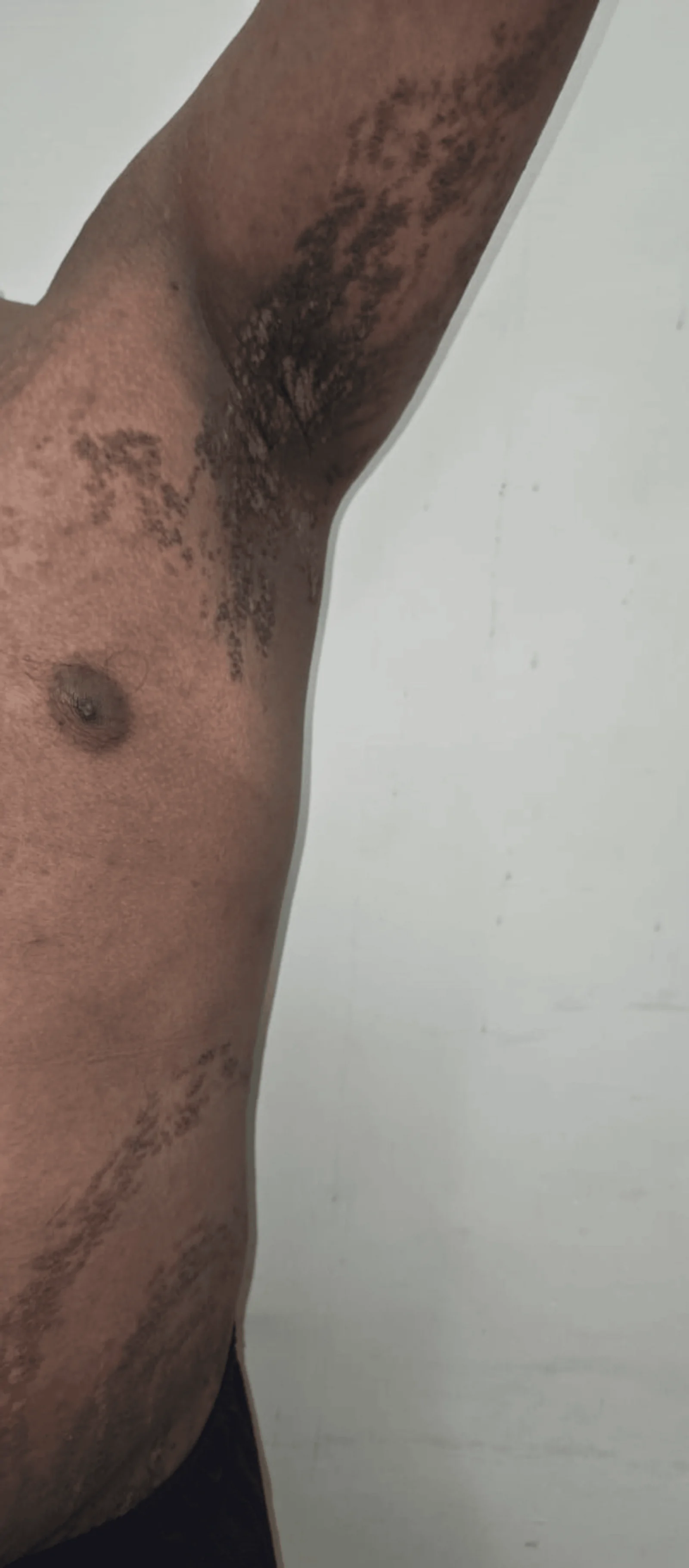
Anterior view showing unilateral hyperpigmented verrucous papules and plaques involving the left upper limb, axilla, chest, and abdomen.

**Figure 2:**
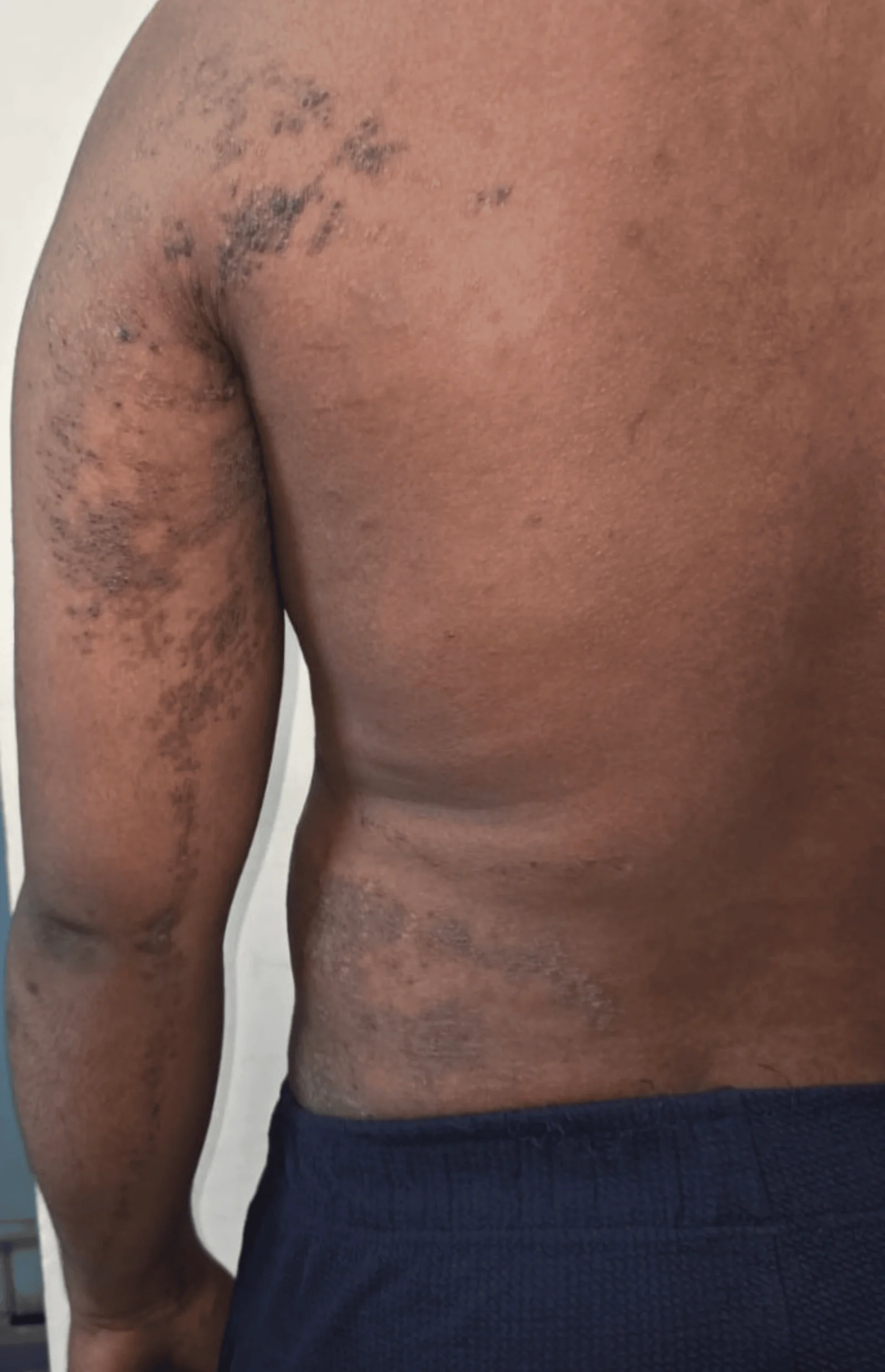
Posterior view of the upper trunk and upper limb showing unilateral hyperpigmented verrucous papules and plaques involving the left shoulder, upper arm, and back.

**Figure 3:**
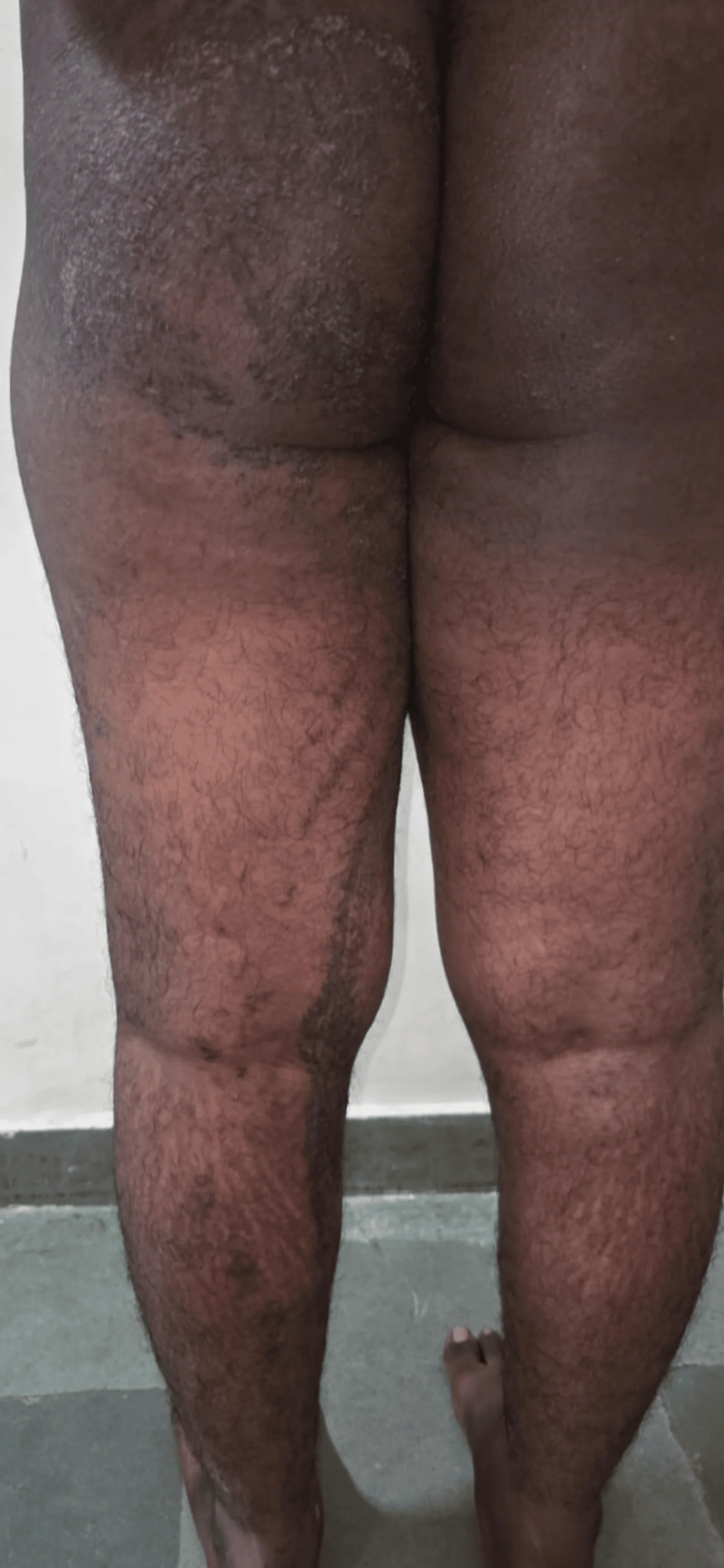
Posterior view showing hyperpigmented verrucous papules and plaques involving the left gluteal region and extending over the posterior thigh and leg.

**Figure 4:**
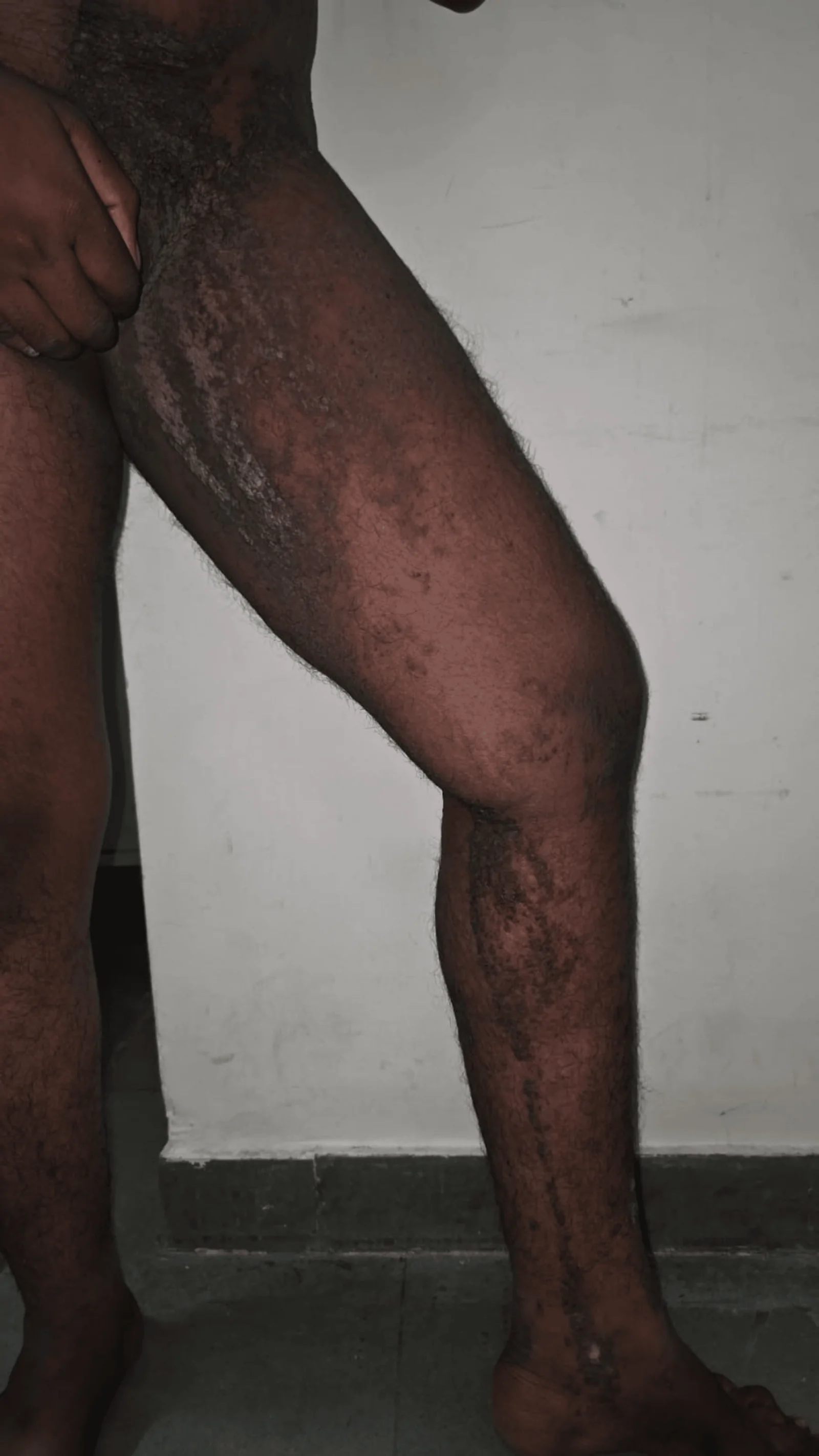
Linear band of hyperpigmented verrucous papules and plaques extending from the left thigh to the ankle.

​Cutaneous examination revealed multiple well-defined, hyperpigmented, verrucous papules and plaques arranged in a linear and Blaschkoid pattern. The distribution was strictly unilateral, confined to the left side of the midline. The lesions extended from the left axilla to the forearm and anteriorly across the abdomen (Figure 1). Additional involvement was noted over the lower back (Figure 2), the left gluteal region (Figure 3), and as a prominent linear band extending down the left lower extremity to the ankle (Figure 4). Examination of the nails, mucosa, and scalp was normal.

​In view of the extensive nature of the nevus, a multisystemic screening was performed. Systemic examination of the cardiovascular, respiratory, and central nervous systems revealed no abnormalities. Routine hematological and biochemical investigations were within normal limits. Dedicated ophthalmology and orthopedic consultations were obtained. The ocular examination revealed no evidence of epibulbar choristomas or other abnormalities. Skeletal surveys and clinical orthopedic evaluation ruled out kyphoscoliosis or limb length discrepancies. Molecular analysis for KRT1 and KRT10 mutations was not performed in the present case because of resource limitations, and the discussion regarding the genetic basis is based on previously published literature

**Figure 5:**
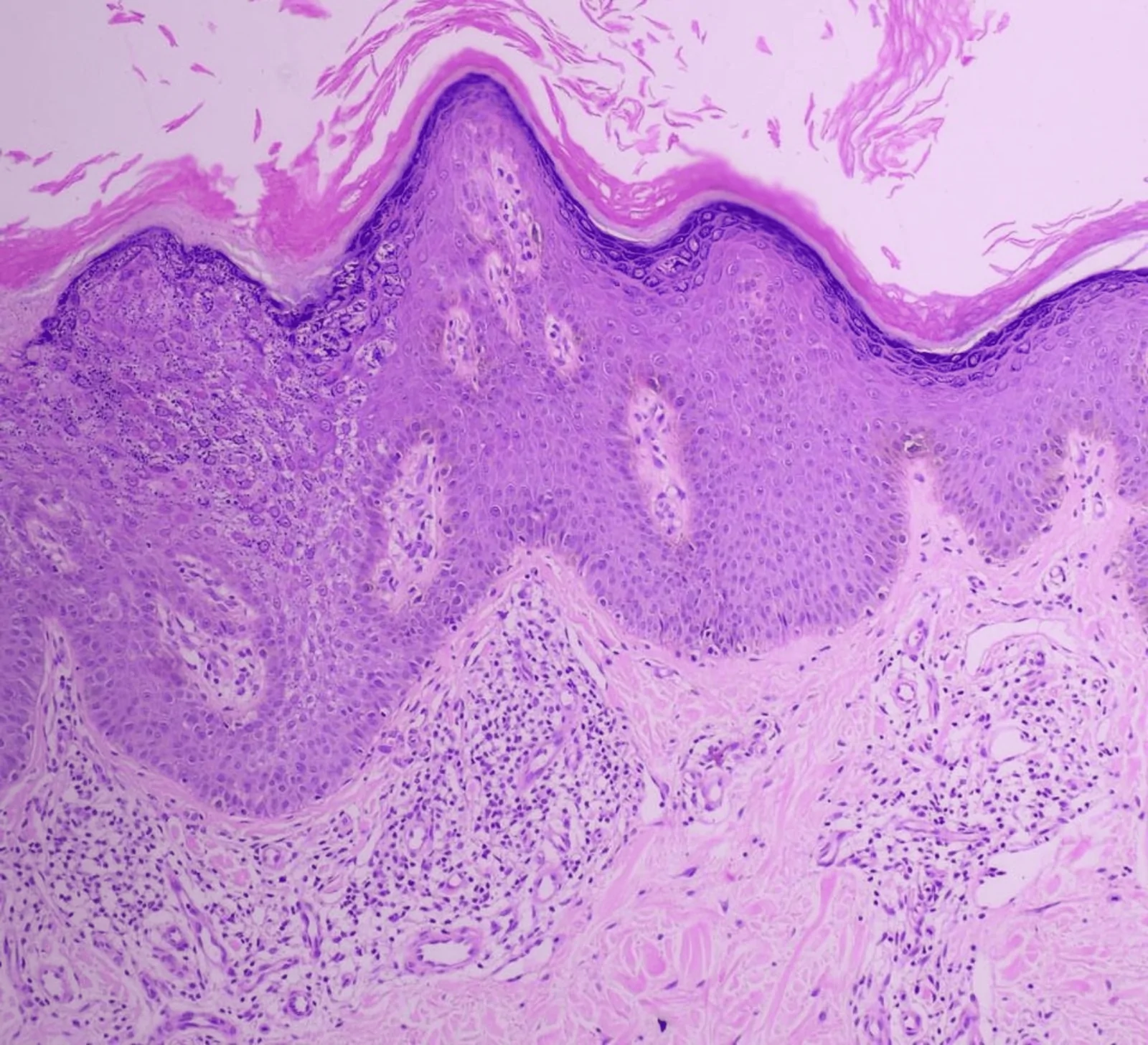
Histopathological examination (hematoxylin and eosin stain, ×200) showing marked orthokeratotic hyperkeratosis, irregular acanthosis, and papillomatosis.

**Figure 6:**
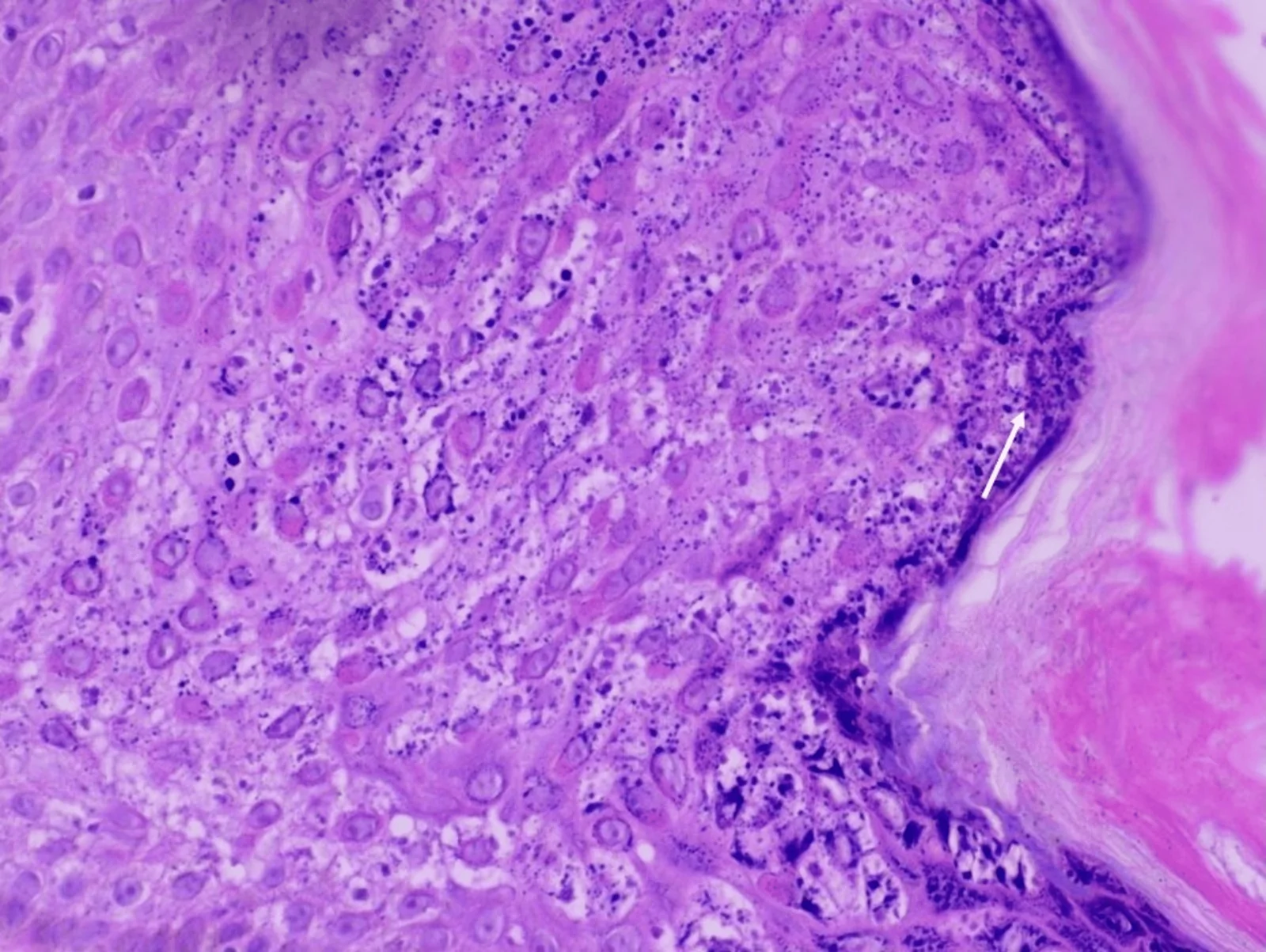
Histopathological examination (hematoxylin and eosin stain, ×400), higher-power view showing epidermolytic (granular) degeneration with coarse basophilic keratohyalin granules (white arrow).

​A punch biopsy was performed from a representative lesion on the left thigh. Histopathological examination of the lesion on the left thigh revealed a verrucous epidermis characterized by marked orthokeratotic hyperkeratosis, irregular acanthosis, and papillomatosis (Figure 5). The diagnostic hallmark was the presence of granular degeneration (epidermolytic hyperkeratosis) involving the stratum granulosum and upper stratum spinosum. This was characterized by prominent perinuclear vacuolization and the presence of coarse basophilic keratohyalin granules​ (Figure 6). These features confirmed the diagnosis of an epidermal nevus with epidermolytic hyperkeratosis.

**Figure 7:**
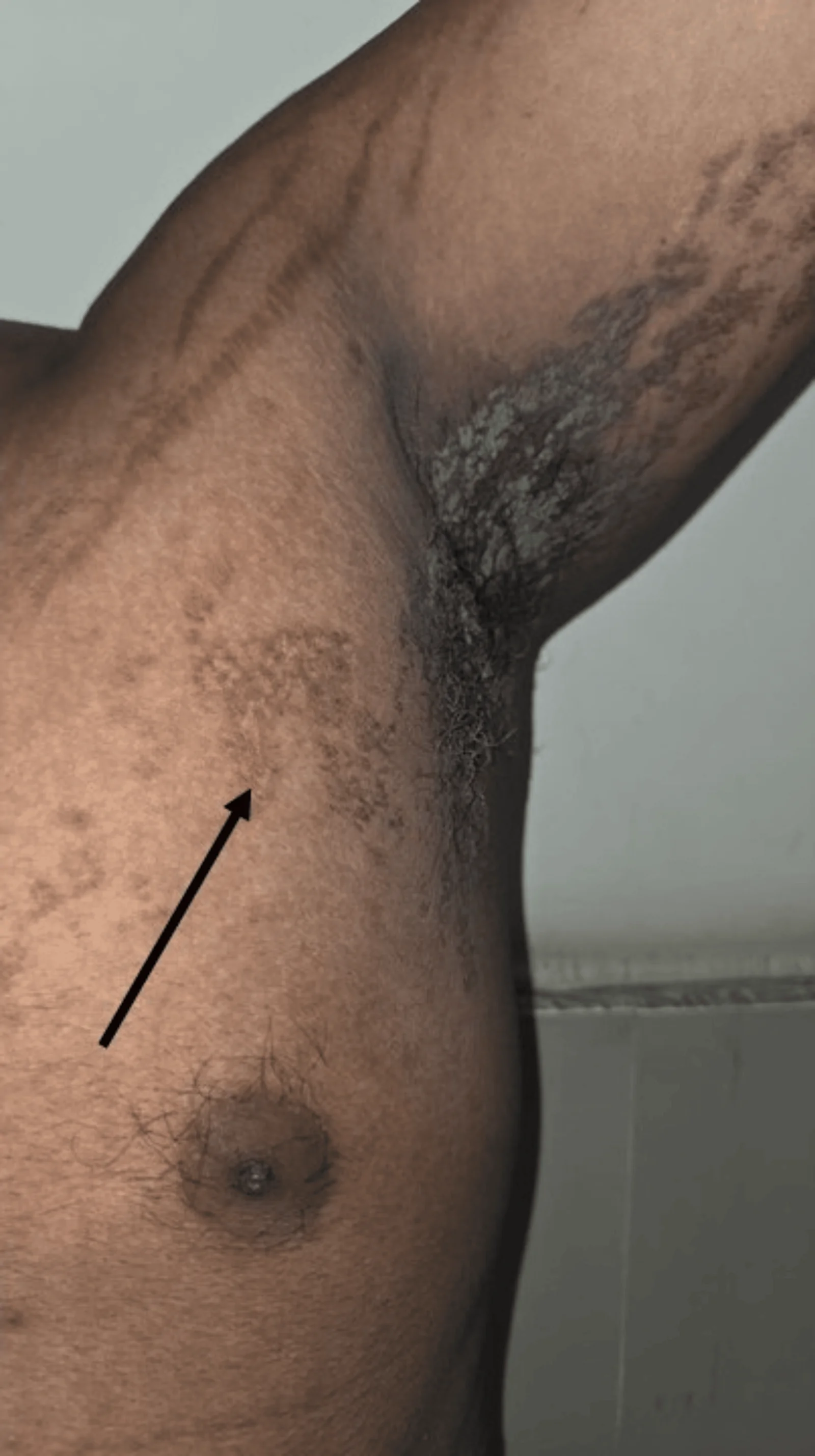
Nine-month follow-up of the left chest lesion after radiofrequency ablation, demonstrating marked flattening of the treated verrucous plaque over the left chest (black arrow) with residual post-inflammatory hyperpigmentation.

**Figure 8:**
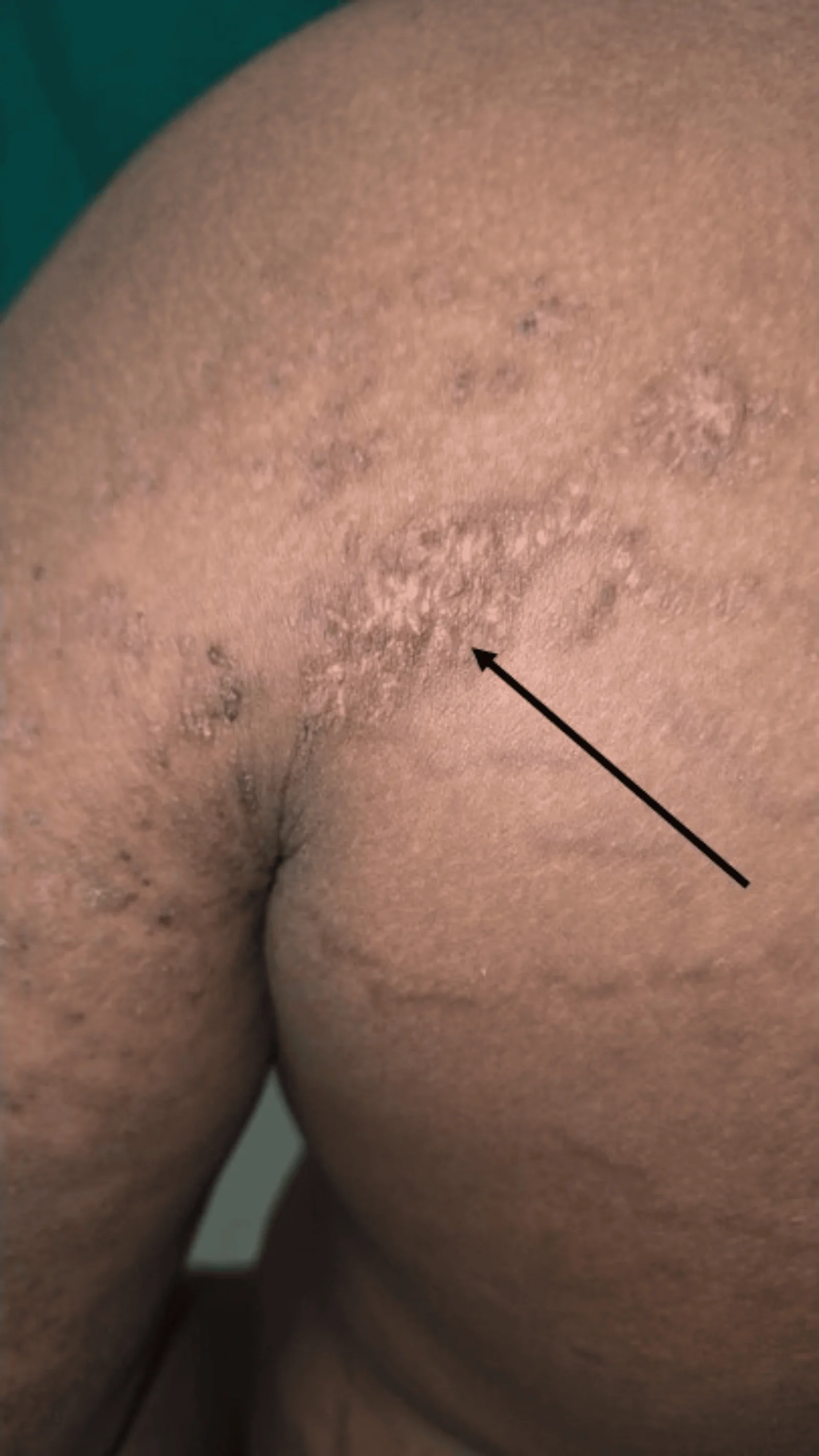
Nine-month follow-up of the posterior shoulder lesion after radiofrequency ablation, demonstrating marked flattening of the treated verrucous plaque over the left posterior shoulder (black arrow) with residual post-inflammatory hyperpigmentation.

​Management focused on cosmetic improvement and the reduction of lesion thickness. The patient underwent two sessions of radiofrequency ablation under local infiltration anesthesia with 2% lignocaine using the Megasurg Gold radiofrequency surgical unit (Dermaindia, Chennai, India). A diamond-shaped radiofrequency electrode was used at a power setting of 2.5 to ablate the lesions. The first session targeted the lesions on the left chest, and a second session targeted lesions over the left upper back. After the procedure, topical mupirocin 2% ointment was prescribed twice daily for one week. The treated areas healed completely within approximately two weeks after each session, with mild residual post-inflammatory hyperpigmentation but no clinically significant scarring. No recurrence was observed in the treated lesions during the nine-month follow-up period (Figures 7, 8).

In view of the potential association with epidermal nevus syndrome, the patient was counseled regarding the need for regular clinical follow-up.

## Discussion

Epidermal nevi are hamartomatous proliferations of the epidermis that arise from post-zygotic somatic mutations leading to genetic mosaicism. The prevalence of epidermal nevi is estimated to be approximately one in 1,000 live births. However, nevus unius lateris is considerably rarer, accounting for only approximately 0.01% of all epidermal nevi [6].

Nevus unius lateris represents the systematized unilateral variant of verrucous epidermal nevus, characterized by extensive hyperkeratotic lesions confined to one side of the body. Common sites include trunk and extremities, while the involvement of the head and neck is relatively uncommon [7]. It results from postzygotic genetic mutations during cutaneous development, producing mosaicism and a characteristic Blaschkoid distribution that follows the embryonic migration pathways of epidermal cells [8]. The lesions may remain stable throughout childhood but can become more prominent during puberty due to hormonal influences and progressive epidermal hyperplasia [9]. The striking unilateral distribution observed in our patient is consistent with this classical presentation.

An important aspect in the evaluation of extensive epidermal nevi is the exclusion of epidermal nevus syndrome, a group of disorders in which cutaneous lesions are associated with extracutaneous abnormalities affecting the central nervous system, skeleton, or eyes [6]. In the present case, a thorough systemic evaluation, including neurological examination, ophthalmological assessment, and orthopedic evaluation, revealed no abnormalities, thereby supporting the diagnosis of an isolated systematized epidermal nevus.

Histopathologically, VEN typically demonstrates hyperkeratosis, acanthosis, and papillomatosis. In some cases, distinctive variants may be observed depending on the underlying genetic mutation [9]. In our patient, prominent epidermolytic hyperkeratosis characterized by coarse basophilic keratohyalin granules and vacuolar degeneration in the upper epidermal layers confirmed the epidermolytic subtype.

Epidermolytic hyperkeratosis represents a characteristic histopathological pattern resulting from mutations in Keratins K1 and K10, which are expressed in the suprabasal layers of the epidermis. These keratins play a crucial role in maintaining the structural integrity of keratinocytes. Mutations in these genes cause suprabasal keratinocyte fragility, resulting in granular degeneration characterized by perinuclear vacuolization, coarse keratohyalin granules, and hyperkeratosis. In cases of epidermal nevus with epidermolytic hyperkeratosis, the mutation usually occurs post-zygotically, resulting in cutaneous mosaicism. Consequently, the abnormal keratinocytes are confined to localized areas of skin, often distributed along Blaschko’s lines. However, if the mutation occurred early enough in embryonic life, some germ cells may also carry the same mutation. Although the parent may present only with localized skin lesions, transmission of a mutated germ cell to the child can cause mutation in all cells, leading to generalized epidermolytic ichthyosis (formerly referred to as bullous congenital ichthyosiform erythroderma) in the child. Therefore, appropriate genetic counseling may be considered in selected patients [9,10].

Management of extensive epidermal nevi is challenging because of the large surface area involved and the risk of recurrence. Conservative therapies, including topical keratolytics, retinoids, and corticosteroids, generally provide limited benefit in thick verrucous lesions. Surgical excision may be associated with significant scarring, particularly in extensive disease. Various ablative modalities, including carbon dioxide laser and radiofrequency ablation, have been used with variable success. Radiofrequency ablation provides controlled removal of hyperkeratotic tissue with favorable cosmetic outcomes, although recurrence may occur [11].

In this patient, radiofrequency ablation was selected as the primary treatment modality due to its easy availability, cost-effectiveness, and precision. Serial treatment sessions resulted in a noticeable reduction in lesion thickness with satisfactory cosmetic improvement and uneventful healing.

## Conclusions

This case highlights an isolated presentation of nevus unius lateris with epidermolytic hyperkeratosis in an adolescent male. Thorough systemic evaluation is essential to exclude associated syndromic abnormalities, while radiofrequency ablation may represent a useful therapeutic option for selected lesions in patients with nevus unius lateris. However, longer follow-up and additional studies are required to determine long-term efficacy and recurrence rates. Furthermore, patients with extensive epidermal nevi should undergo long-term clinical follow-up to monitor for the development of syndromic manifestations. In addition, epidermolytic epidermal nevi may rarely be associated with gonadal mosaicism; therefore, genetic counseling should be considered in selected patients, especially those of reproductive age.
